# Bulk superconductivity in a four-layer-type Bi-based compound La_2_O_2_Bi_3_Ag_0.6_Sn_0.4_S_5.7_Se_0.3_

**DOI:** 10.1038/s41598-019-49934-z

**Published:** 2019-09-19

**Authors:** Rajveer Jha, Yosuke Goto, Tatsuma D. Matsuda, Yuji Aoki, Masanori Nagao, Isao Tanaka, Yoshikazu Mizuguchi

**Affiliations:** 1 Department of Physics, Tokyo Metropolitan University, 1-1 Minami-Osawa, Hachioji, Tokyo 192-0397 Japan; 2 University of Yamanashi, 7-32, Miyamae, Kofu, Yamanashi 400-8511 Japan

**Keywords:** Superconducting properties and materials, Phase transitions and critical phenomena

## Abstract

Recently, we reported the observation of superconductivity at ~0.5 K in a La_2_O_2_M_4_S_6_-type (M: metal) layered compound La_2_O_2_Bi_3_AgS_6_, which is a layered system related to the BiS_2_-based superconductor system but possesses a thicker Bi_3_AgS_6_-type conducting layer. In this study, we have developed the La_2_O_2_Bi_3_AgS_6_-type materials by element substitutions to increase the transition temperature (*T*_c_) and to induce bulk nature of superconductivity. A resistivity anomaly observed at 180 K in La_2_O_2_Bi_3_AgS_6_ was systematically suppressed by Sn substitution for the Ag site. By the Sn substitution, *T*_c_ increased, and the shielding volume fraction estimated from magnetization measurements also increased. The highest *T*_c_ (=2.3 K) and the highest shielding volume fraction (~20%) was observed for La_2_O_2_Bi_3_Ag_0.6_Sn_0.4_S_6_. The superconducting properties were further improved by Se substitutions for the S site. By the combinational substitutions of Sn and Se, bulk-superconducting phase of La_2_O_2_Bi_3_Ag_0.6_Sn_0.4_S_5.7_Se_0.3_ with a *T*_c_ of 3.0 K (*T*_c_^onset^ = 3.6 K) was obtained.

## Introduction

Layered superconductors have been extensively studied due to observations of unconventional superconductivity and high transition temperature (*T*_c_)^1,2^. In addition, the great flexibility of constituent elements and layered (stacking) structure, the studies on layered superconductor system can be widely developed. The recent discovery of BiS_2_-based superconductors has also created remarkable attention in the superconductivity community: the typical materials are Bi_4_O_4_S_3_, REO_1−x_F_x_BiS_2_ (RE = La, Ce, Pr, Nd, Yb), and Sr_1−*x*_RE_*x*_FBiS_2_^3–14^. For instance, the crystal structure of the typical system LaOBiS_2_ is composed of alternate stacks of a La_2_O_2_ blocking layer and two BiS_2_ layers. Since the parent phase (LaOBiS_2_) is an insulator with a band gap^15^, electron doping is needed to induce metallic and superconducting characteristics. Furthermore, local in-plane structure should be optimized to induce bulk superconductivity in the BiS_2_ layer^16^. Thus, the *T*_c_ of BiS_2_-based is sensitive to physical pressure^4,17–21^ and chemical pressure^22–24^ effects and reaches 11 K in LaO_0.5_F_0.5_BiS_2_. Therefore, an increase in the highest record of *T*_c_ in the BiS_2_-based superconductor family can be expected by further material development and tuning of structural and electrical properties^14^.

**Figure 1:**
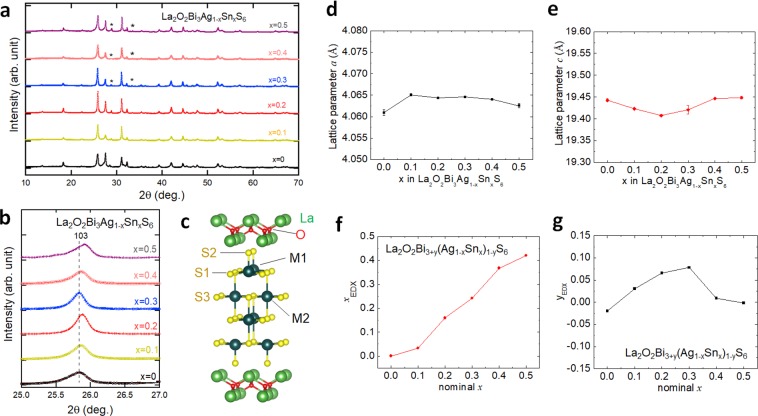
Crystal structure and composition analyses results for La_2_O_2_Bi_3_Ag_1−*x*_Sn_*x*_S_6_ (*x* = 0–0.5). (**a**) Room-temperature XRD pattern for *x* = 0–0.5. The impurity peaks of La_2_Sn_2_O_7_ are indicated by asterisks (*). (**b**) XRD pattern near the 103 (Miller index) peak of the tetragonal phase of La_2_O_2_Bi_3_AgS_6_. (**c**) A schematic image of the crystal structure of La_2_O_2_M_4_S_6_ (two M sites, M1 and M2, are occupied by Bi, Ag, and Sn in the present system). (**d**,**e**) Lattice parameters of *a* and *c* obtained from Rietveld refinements. (**f**,**g**) Nominal composition dependences of compositions (*x* and *y*) analyzed by EDX, where *x* and *y* are defined as La_2_O_2_Bi_3+*y*_(Ag_1−*x*_Sn_*x*_)_1−*y*_S_6_.

Very recently, we reported on the superconductivity in oxychalcogenide La_2_O_2_Bi_3_AgS_6_ with *T*_c_ of 0.5 K^25^. The crystal structure of La_2_O_2_Bi_3_AgS_6_ is similar to the typical BiS_2_-based superconductor LaOBiS_2_, but the conducting layer thickness is thicker than that of LaOBiS_2_. The crystal structure of the system can be described as La_2_O_2_M_4_S_6_, in which the M site can be Pb, Bi, and Ag^26–28^. There are two different metal sites [M1 and M2 displayed in Fig. 1(c)] in the M_4_S_6_-type conducting layer. For the outer layers with the M1 site, Bi selectively occupies, and the layers can be regarded as the BiS_2_-type layers. For the inner layer with the M2 site, NaCl-type (Bi,Ag)S_2_ layers are inserted in between the BiS_2_-type layers.

Interestingly, an anomalous transport property was observed in the temperature dependence of resistivity in La_2_O_2_Bi_3_AgS_6_^25^, which is similar to the charge-density-wave (CDW) transition in EuFBiS_2_^29^. In this study, we have investigated the Sn substitution effect for the Ag site in La_2_O_2_Bi_3_Ag_1−*x*_Sn_*x*_S_6_ and found that the resistivity anomaly was suppressed by Sn substitutions. *T*_c_ reached 2.3 K for *x* = 0.4 (La_2_O_2_Bi_3_Ag_0.6_Sn_0.4_S_6_). Furthermore, by Se substitutions for the S site in La_2_O_2_Bi_3_Ag_0.6_Sn_0.4_S_5.7_Se_0.3_, the *T*_c_ further increased to 3.5 K, and bulk nature of superconductivity was confirmed from magnetic shielding volume fraction.

## Results

### Sn substitution effect on structural and physical properties in La_2_O_2_Bi_3_Ag_1−*x*_Sn_*x*_S_6_

Figure 1a displays the room temperature XRD patterns for La_2_O_2_Bi_3_Ag_1−*x*_Sn_*x*_S_6_ (*x* = 0–0.5). All the La_2_O_2_Bi_3_Ag_1−*x*_Sn_*x*_S_6_ samples are crystallized in the tetragonal structure with the space group of *P*4/*nmm*. An impurity phase of La_2_Sn_2_O_7_ was observed for *x* = 0.2–0.5. Figure 1b shows the shift in the 103 peak position, which slightly shifts towards the low angle side for *x* = 0.1. As the Sn concentration increases from *x* = 0.2 to 0.5, the 103 peak shifts towards the higher angle side. The schematic image of the crystal structure of La_2_O_2_M_4_S_6_ is shown in Fig. 1c. For the La_2_O_2_Bi_3_Ag_1−*x*_Sn_*x*_S_6_ phase, Bi selectively occupies the M1 site, and Sn is expected to substituted for Ag at the M2 site, which was qualitatively confirmed by Rietveld refinements. The evolutions of the lattice parameters by the Sn substitution are shown in Fig. 1d,e. The lattice parameters are *a* = 4.061(1) Å and *c* = 19.445(1) Å for *x* = 0 and *a* = 4.0648(1) Å and *c* = 19.48(1) Å for *x* = 0.1. The lattice parameter *c* tends to decrease by Sn substitution for *x* = 0–0.2, and then, it continuously increases with increasing *x* for *x* = 0.2–0.5. The lattice parameter *a* increases for the *x* = 0.1, but it tends to decrease with increasing *x* for higher *x*. However, the changes in those lattice parameters are small. On the basis of the lattice parameter evolutions, we consider that the Sn substitution does not largely affect the lattice volume, which may be due to smaller ionic radius of Sn^2+^ (93 pm) than that of Ag^+^ (113 pm). The La_2_O_2_Bi_3_AgS_6_-type structure can be considered as the stacking of a La_2_O_2_ layer, two BiS_2_ layers, and an NaCl-type AgBiS_2_ layer. In such a layered structure, the unit cell is almost determined by the hardest layer due to the different ionic bonding nature. In this structure, La_2_O_2_ layer structure is the hardest.

The actual ratio of the metals in the conducting layer (Bi, Ag, and Sn) in the La_2_O_2_Bi_3_Ag_1−*x*_Sn_*x*_S_6_ (*x* = 0–0.5) samples were examined by EDX. The nominal and analyzed values of *x* are plotted in the Fig. 1f. Although there is slight deviation from the nominal values, the analyzed values of *x*_EDX_ linearly increases with increasing nominal *x*. We found that Bi is slightly excess for *x* = 0.1–0.3. Therefore, *y* parameter with a formula of La_2_O_2_Bi_3+*y*_(Ag_1−*x*_Sn_*x*_)_1−*y*_S_6_ was introduced to analyze the Bi concentration. The analyzed values of *y*_EDX_ are plotted in Fig. 1g. *y*_EDX_ is higher for *x* = 0.1–0.3 but almost zero for *x* = 0, 0.4, and 0.5. Therefore, we consider that the Bi excess can be ignored in the discussion on the Sn substitution (and Se substitution) effect, and we use the formula La_2_O_2_Bi_3_Ag_1−*x*_Sn_*x*_S_6_ in this paper.

**Figure 2:**
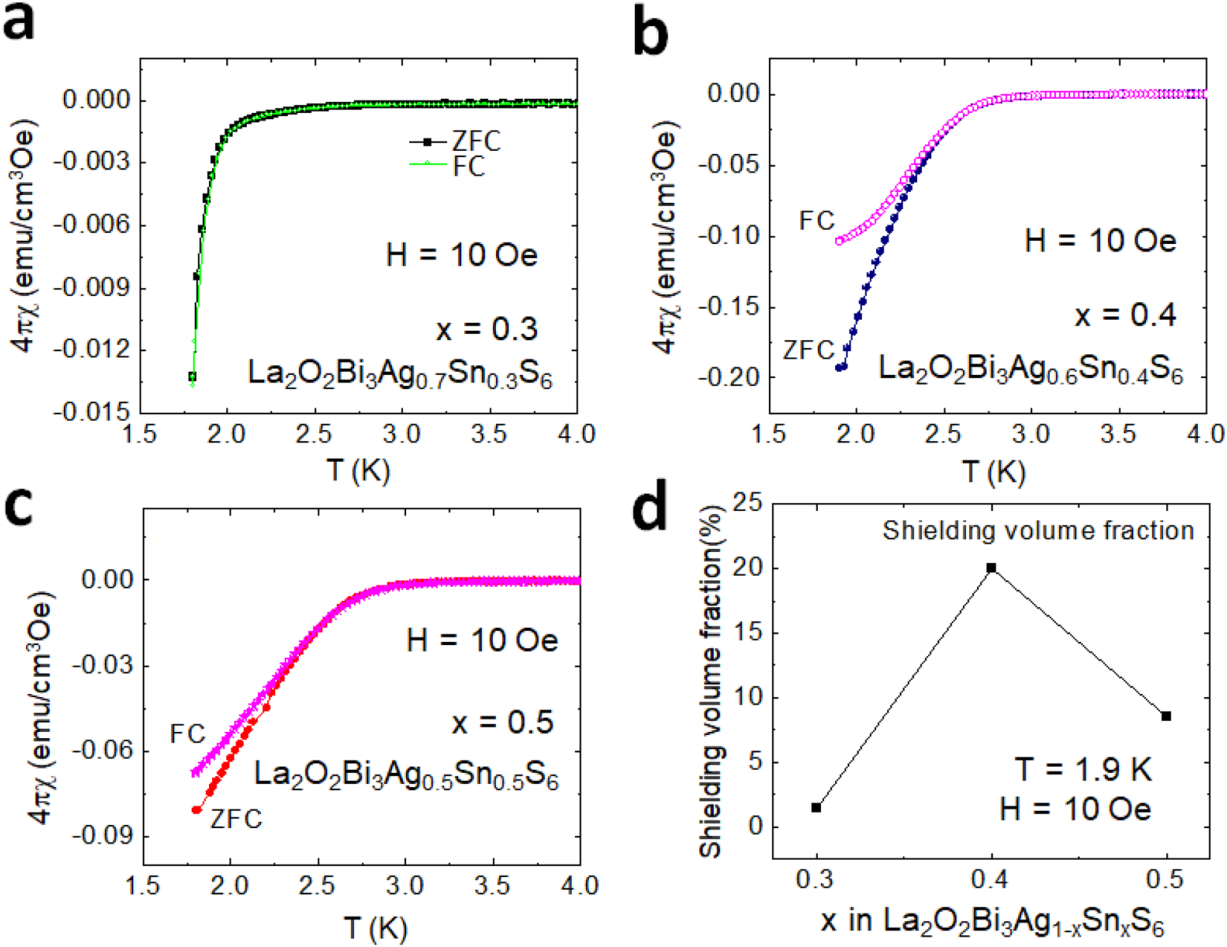
Superconducting properties examined from magnetic susceptibility for La_2_O_2_Bi_3_Ag_1−*x*_Sn_*x*_S_6_ (*x* = 0.3–0.5). (**a**–**c**) Temperature (*T*) dependences of magnetic susceptibility (4π*χ*) for *x* = 0.3–0.5 measured in the ZFC and FC modes with an applied magnetic field of 10 Oe. (**d**) Sn concentration dependence of the shielding volume fraction estimated using the ZFC data at 1.9 K.

Figure 2a–c show the temperature dependences of magnetic susceptibility (4π*χ*-*T*) under an applied magnetic field of 10 Oe for La_2_O_2_Bi_3_Ag_1−*x*_Sn_*x*_S_6_ (*x* = 0.3–0.5). The diamagnetic signals in the 4π*χ* curve were observed below 2.2, 2.8, and 2.6 K for *x* = 0.3, 0.4, and 0.5, respectively. A large diamagnetic signal was observed below 2.8 K in the ZFC curve for *x* = 0.4. The shielding value fractions estimated from 4π*χ* (ZFC) at 1.9 K is nearly 20% [See Fig. 2(d).] while it is still not saturated. From the susceptibility results, we consider that Sn substitution is effective to improve the superconducting properties of La_2_O_2_Bi_3_AgS_6_ but not sufficient to induce bulk superconductivity.

**Figure 3:**
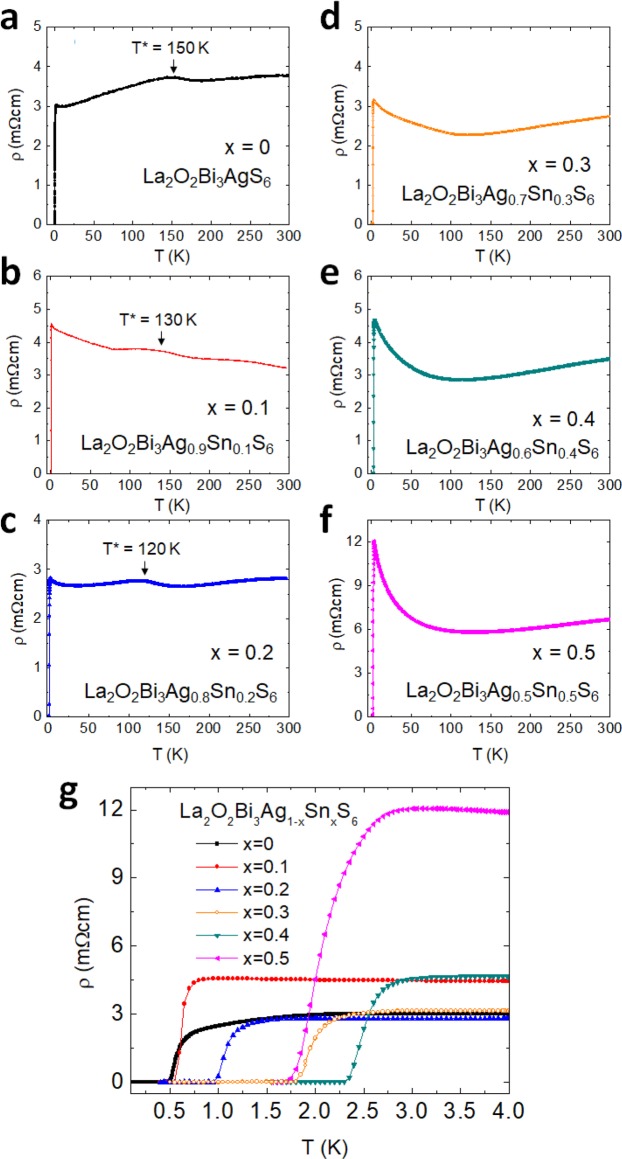
Electrical transport properties for La_2_O_2_Bi_3_Ag_1−*x*_Sn_*x*_S_6_ (*x* = 0–0.5). (**a**–**f**) Temperature dependences of electrical resistivity from 300 to 0.1 K for *x* = 0–0.5. The anomaly temperature in the *ρ*(*T*) curves is indicated by T*. (**g**) The *ρ*(*T*) curves in the temperature range of 0.1–4.0 K.

Figure 3 shows the temperature dependences of electrical resistivity from 300 to 0.1 K for La_2_O_2_Bi_3_Ag_1−*x*_Sn_*x*_S_6_ (*x* = 0–0.5). The electrical resistivity at 300 K decreases with increasing Sn concentration up to *x* = 0.3 and increases again for *x* = 0.4 and 0.5. The normal-state resistivity of the Sn-doped samples changes remarkably. For example, the pure sample (*x* = 0) shows a linear decrease in resistivity on cooling below the anomaly temperature T* = 180 K. A similar behavior was observed up to *x* = 0.2. The resistivity anomaly at T* appears for *x* ≤ 0.2, and the T* shifts towards the lower temperature side with increasing *x*. In contrast, the normal-state *ρ*(*T*) for *x* = 0.3–0.5 shows an upturn below ~50 K. The anomaly disappears for *x* ≥ 0.3. Figure 3g shows the zoomed view of the Figs. 3a–f near the superconducting transition. The *T*_c_ clearly increases with increasing Sn concentration in La_2_O_2_Bi_3_Ag_1−*x*_Sn_*x*_S_6_. The highest *T*_c_ was achieved for *x* = 0.4, and *T*_c_ decreases for a higher substitution with *x* = 0.5.

**Figure 4:**
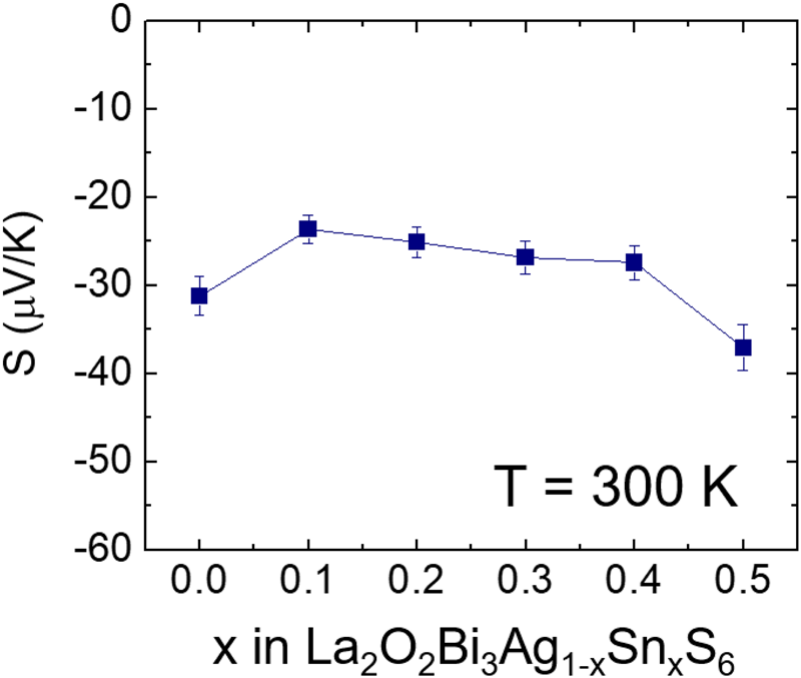
Seebeck coefficient for La_2_O_2_Bi_3_Ag_1−*x*_Sn_*x*_S_6_ (*x* = 0–0.5). The room-temperature Seebeck coefficient (*S*) is plotted as a function of nominal Sn concentration (*x*).

The room-temperature Seebeck coefficient (*S*) for La_2_O_2_Bi_3_Ag_1−*x*_Sn_*x*_S_6_ (*x* = 0–0.5) are shown in Fig. 4. The Seebeck coefficient is a good scale for the carrier concentration in BiS_2_-based compounds^30^. We observed a slight change in *S* by Sn substitution. The *S* in *x* = 0.2–0.4 are almost the same, but that for *x* = 0 and 0.5 are slightly large. This suggests that the carrier concentrations for *x* = 0.2–0.4 are higher than those for *x* = 0 and 0.5. This seems to be related to the evolution of *T*_c_. However, the large change in *T*_c_ from 0.6 to 2.3 K between *x* = 0.1 and 0.4 cannot be simply understood by the carrier concentration only.

**Figure 5:**
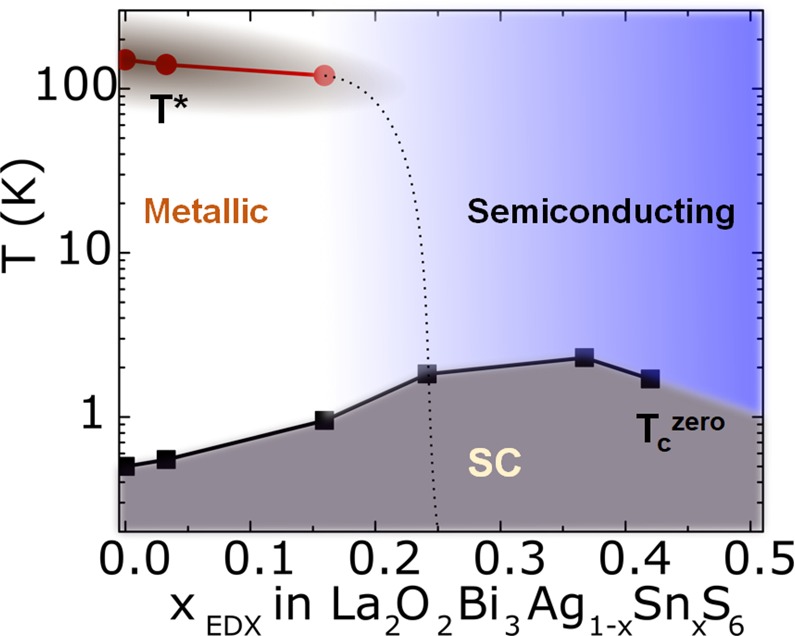
Phase diagram for La_2_O_2_Bi_3_Ag_1−*x*_Sn_*x*_S_6_ (*x* = 0–0.5). The *x* (Sn concentration estimated from EDX) dependences of *T*_c_^zero^ and T^*^ are plotted as a function of Sn concentration (*x*). SC denotes superconductivity.

Figure 5 shows the superconductivity phase diagram of La_2_O_2_Bi_3_Ag_1−*x*_Sn_*x*_S_6_, which shows the interplay between the resistivity anomaly temperature (T^*^) and the superconducting transition temperature (*T*_c_^zero^). The T* is suppressed by the Sn substitution, and it disappears at *x* = 0.3. The *T*_c_ gradually increases with increasing *x* in La_2_O_2_Bi_3_Ag_1−*x*_Sn_*x*_S_6_. The highest *T*_c_^zero^ = 2.3 K is achieved for *x* = 0.4. A lower *T*_c_^zero^ = 1.9 K is observed for the highest (solubility-limit) Sn concentration of *x* = 0.5.

Here, we discuss about the possible influence of the presence of the La_2_Sn_2_O_7_ impurity to the composition. Due to the change in the impurity amount for *x* = 0–0.5, the actual compositions may deviate from the nominal compositions. Although the Sn concentration to that of Ag was checked by EDX (Fig. 1f), oxygen deficiency in the blocking layer was not checked in this study. However, we consider that oxygen deficiency was not introduced because it is expected to make the structure unstable even if it has been introduced in the La_2_O_2_Bi_3_AgS_6_-type structure. We have tried to dope electrons by oxygen deficiency or fluorine substitution for the La_2_O_2_Bi_3_AgS_6_-type structure. However, in the La_2_O_2_Bi_3_AgS_6_ system, such trials of fluorine substitutions (or oxygen deficiency) resulted in decomposing of the La_2_O_2_Bi_3_AgS_6_-type structure into LaO_1−*x*_F_*x*_BiS_2_. This indicates that an electron-doped composition with the La_2_O_2_Bi_3_AgS_6_-type structure cannot be obtained easily due to the competition to the high stability of the REO_1−*x*_F_*x*_BiS_2_-type phase. In addition, oxygen deficiency has not been observed in the REOBiS_2_ systems.

### Superconducting properties of Se-doped La_2_O_2_Bi_3_Ag_0.6_Sn_0.4_S_5.7_Se_0.3_

As shown above, the Sn substitution improved the superconducting properties in La_2_O_2_Bi_3_Ag_1−*x*_Sn_*x*_S_6_, and the highest *T*_c_ and shielding volume fraction were obtained for *x* = 0.4. In the BiS_2_-based compounds, partial Se substitutions for the S site of the superconducting BiS_2_ layers have significantly improved the superconducting properties and the bulk characteristics of superconductivity. Therefore, we tried to substitute the S site  by Se for the *x* = 0.4 sample. The 5%-Se sample La_2_O_2_Bi_3_Ag_0.6_Sn_0.4_S_5.7_Se_0.3_ was successfully synthesized, but samples with higher Se concentration contained selenide impurity phases. The solubility limit of Se for the S site is around 5%. The composition estimated from the EDX analyses for Bi, Ag, Sn, S, Se elements was La_2_O_2_Bi_3.09_Ag_0.65_Sn_0.26_S_5.73_Se_0.27_. Since the obtained composition is close to the nominal formula, we call the sample with the nominal value below.

**Figure 6:**
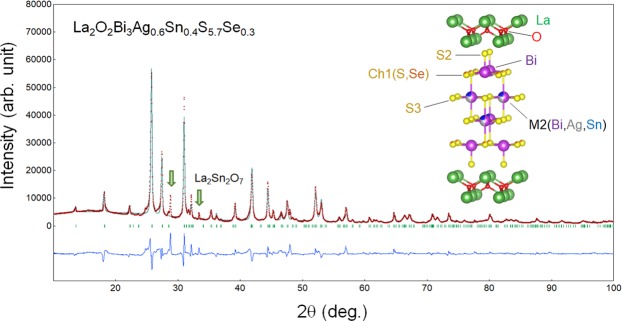
X-ray diffraction analysis for La_2_O_2_Bi_3_Ag_0.6_Sn_0.4_S_5.7_Se_0.3_. XRD pattern and the Rietveld refinement result are shown. The arrows indicate the peaks for the impurity phase La_2_Sn_2_O_7_. The inset image shows the crystal structure depicted using the structural parameters obtained from the Rietveld refinement.

Figure 6 shows the XRD pattern and the Rietveld refinement result for La_2_O_2_Bi_3_Ag_0.6_Sn_0.4_S_5.7_Se_0.3_. Although two peaks related to the La_2_Sn_2_O_7_ impurity phase were observed, other peaks could be refined using the tetragonal (*P*4/*nmm*) model with a reliability factor *R*_wp_ of 13.4%. In the refinement, Se was assumed to be substituted for the S1 site. The lattice parameters were *a* = 4.0759(2) Å and 19.4824(11) Å, which are clearly larger than those of La_2_O_2_Bi_3_Ag_1−*x*_Sn_*x*_S_6_ due to the presence of Se.

**Figure 7:**
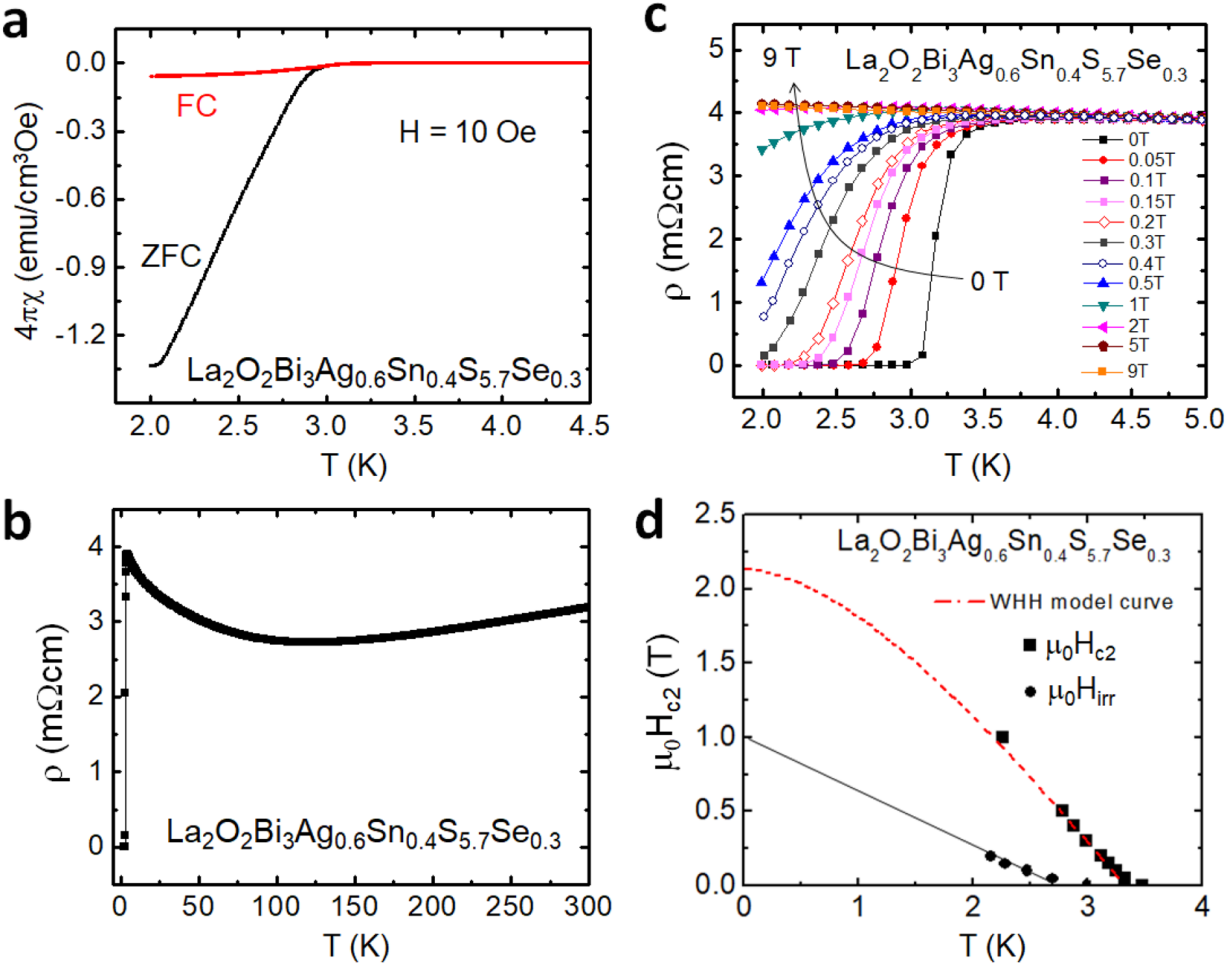
Superconducting properties for La_2_O_2_Bi_3_Ag_0.6_Sn_0.4_S_5.7_Se_0.3_. (**a**) Temperature dependence of magnetic susceptibility. (**b**) Temperature dependence of electrical resistivity [*ρ*(*T*)]. (**c**) Low-temperature *ρ*(*T*) under magnetic fields up to 9T. (**d**) Temperature-magnetic field phase diagram with the upper critical field (*H*_c2_) and the irreversible field (*H*_irr_).

Figure 7 displays the superconducting properties of La_2_O_2_Bi_3_Ag_0.6_Sn_0.4_S_5.7_Se_0.3_. As shown in Fig. 7a, a large shielding volume fraction close to 100% was observed. From the resistivity measurements (Fig. 7b), zero resistivity was observed at 3.0 K, and the onset temperature (*T*_c_^onset^) was 3.5 K; we estimated the temperature where the resistivity becomes almost 90% of normal-state resistivity. Although superconductivity was observed, the *ρ*(*T*) curve still shows a semiconducting-like localization at low temperatures. We have measured *ρ*(*T*) under magnetic fields up to 9 T. The obtained *T*_c_^onset^ and *T*_c_^zero^ were plotted in Fig. 7d to evaluate the upper critical field *H*_c2_ and the irreversible field *H*_irr_. The *H*_c2_(0) was estimated as 2.15 T using the WHH model (Werthamer-Helfand-Hohenberg model)^31^. In addition, from rough estimation with a linear fitting of *H*_irr_, the *H*_irr_(0) was estimated as 1.0 T.

## Discussion

### Suppression of resistivity anomaly by the Sn substitution

Here, we discuss the possible origin of the increase in *T*_c_ by the Sn substitution. As revealed in the crystal structure part, the lattice parameters were not largely affected by the Sn substitution. Therefore, in-plane chemical pressure amplitude in the Bi-S superconducting plane, which has been revealed as the essential parameter for the emergence of superconductivity in BiS_2_-based compounds^24^, should not be significantly changed. Therefore, we consider that the in-plane chemical pressure effect is not the origin for the increase in *T*_c_ by the Sn substitution. On carrier concentration, the absolute value of the Seebeck coefficient slightly decreases by Sn substitution for *x* = 0.1–0.4, which can be corresponding to the slight increase in electron carriers by Sn substitution. However, the large increase in *T*_c_ for *x* = 0.4 may not be understood from the increase in carrier concentration only because the difference in carrier concentration between *x* = 0.1 (*T*_c_ = 0.6 K) and *x* = 0.4 (*T*_c_ = 2.3 K) is expected to be quite small. On the basis of these facts, we briefly mention about the possible relation to the CDW ordering and the possible scenario of the suppression of CDW by Sn substitution in this system. In the *ρ*-*T* plots, an anomaly was observed in the La_2_O_2_Bi_3_Ag_1−*x*_Sn_*x*_S_6_ system. A similar feature in the normal-state resistivity has been observed in the EuFBiS_2_ superconductor (*T*_c_ = 0.3 K). The origin of the hump was proposed as a CDW transition^29^. We assume that the suppression of the CDW ordering is the origin for the increased *T*_c_ in La_2_O_2_Bi_3_Ag_1−*x*_Sn_*x*_S_6_. In addition, the anomaly temperature T^*^ was shifted to a lower temperature by Sn substitution, and the anomaly disappeared at *x* = 0.3. At around *x* = 0.3 and 0.4, *T*_c_ is the maximum. These facts imply that *T*_c_ increased by the suppression of T^*^. Although we have no evidence for the CDW states in the La_2_O_2_Bi_3_Ag_1−*x*_Sn_*x*_S_6_ system and the suppression mechanism by the Sn substitution, introduction of randomness at the M2 site may be effective to suppress the charge ordering states.

### Bulk superconductivity in La_2_O_2_Bi_3_Ag_0.6_Sn_0.4_S_5.7_Se_0.3_

As shown in the Result part, a partial Se substitution for S induced bulk superconductivity in La_2_O_2_Bi_3_Ag_0.6_Sn_0.4_S_5.7_Se_0.3_. Although the solubility limit of Se for the S site is very low (5%), the lattice parameters clearly changed by the partial Se substitution, and the superconducting properties were significantly improved. Although we refined three models with different Se site (assuming the substitution at the S1, S2, or S3 sites), we could not find the site selectivity of doped Se. However, we expect that the doped Se occupies the Ch1 site in the inset of Fig. 6. In previous reports on the Se substitution in BiS_2_-based compounds, the site selectivity of Se at the in-plane site was observed^24,32,33^. According to the relationship between in-plane disorder at the chalcogen site and superconductivity in BiS_2_-based systems^16,24^, we assume that Se substitution reduced the in-plane disorder at the S1 site and induced bulk superconductivity. Furthermore, the room-temperature Seebeck coefficient for the La_2_O_2_Bi_3_Ag_0.6_Sn_0.4_S_5.7_Se_0.3_ sample was similar to those shown in Fig. 4 (*S* = −25 μV/K). This also suggests that the bulk nature of superconductivity was induced by local structural optimization but not due to changes in carrier concentration.

Since the phases contain Sn in their structure, we have to exclude the possibility of superconductivity of elemental Sn in the examined samples. From XRD, no peaks of Sn was observed. However, amorphous Sn may exist and show superconducting transition. We cannot directly exclude this possibility, but we consider that the bulk superconductivity observed here is not originated from elemental Sn. We have investigated similar Sn substitution effects in the La_2_O_2_Bi_2_Pb_2−*x*_Sn_*x*_S_6_ system. La_2_O_2_Bi_2_Pb_2_S_6_ (LaOBiPbS_3_) shows an insulating transport property^26,27^. The insulating behavior was not suppressed by Sn substitution, and superconducting transition was not observed for all the examined samples of La_2_O_2_Bi_2_Pb_2−*x*_Sn_*x*_S_6_. This fact may indirectly suggest that the bulk superconductivity observed in the La_2_O_2_Bi_3_Ag_0.6_Sn_0.4_S_5.7_Se_0.3_ sample is not caused by Sn amorphous impurities.

**Figure 8:**
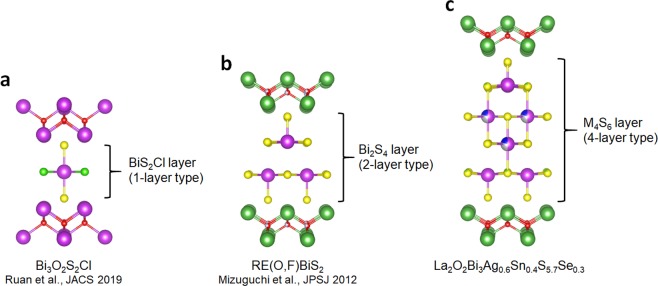
Comparison of crystal structure of typical Bi-based layered compounds with different thickness of superconducting layer. (**a**) Schematic image of the crystal structure of Bi_3_O_2_S_2_Cl, which was recently discovered by Ruan *et al*.^34^. In this material, the conducting layer can be regarded as the one-layer-type BiS_2_Cl. (**b)** Schematic image of the crystal structure of the typical BiS_2_-based superconductor RE(O,F)BiS_2_. In the series, the conducting layer can be regarded as the two-layer-type Bi_2_S_4_ bilayer. (**c)** Schematic image of the crystal structure of La_2_O_2_Bi_3_AgS_6_-type (La_2_O_2_M_4_S_6_-type) materials whose conducting layer can be regarded as the four-layer-type M_4_S_6_ layer.

In conclusion, we have reported the emergence of bulk superconductivity in La_2_O_2_M_4_S_6_-type (four-layer-type) layered oxychalcogenide La_2_O_2_Bi_3_Ag_0.6_Sn_0.4_S_5.7_Se_0.3_. According to the material design strategy shown here, we can further develop related four-layer-type Bi-based layered superconductors. Recently, a new superconductor Bi_3_O_2_S_2_Cl with the one-layer-type Bi-Cl-S superconducting layer was discovered by Ruan *et al*.^34^. In Fig. 8, schematic images of typical one-layer-type (Fig. 8a), two-layer-type (Fig. 8b), and four-layer-type (Fig. 8c) are displayed for comparison. All the materials have the similar RE_2_O_2_ or Bi_2_O_2_ blocking layer. By changing the constituent elements in the superconducting layers, the thickness can be changed in this superconductor family. On the basis of these facts, we will be able to design various types of Bi-based layered superconductors with a higher *T*_c_ as developed with various Cu-oxide layers in the cuprate family. With such material development with a different type of conducting layer, remarkable changes in superconducting properties can be expected.

## Methods

The polycrystalline samples of La_2_O_2_Bi_3_Ag_1−*x*_Sn_*x*_S_6_ with *x* = 0, 0.1, 0.2, 0.3, 0.4, and 0.5 were prepared by a solid-state reaction method. The polycrystalline samples of Se-substituted La_2_O_2_Bi_3_Ag_0.6_Sn_0.4_S_6−*z*_Se_*z*_ with *z* = 0.3 and 0.6 were also prepared by a solid-state reaction method. Powders (or grains) of Bi_2_O_3_ (99.9%), La_2_S_3_ (99.9%), Sn (99.99%), and AgO (99.9%) and grains of Bi (99.999%), S (99.99%), and Se (99.99%) with a nominal composition of La_2_O_2_Bi_3_Ag_1−*x*_Sn_*x*_S_6−*z*_Se_*z*_ were mixed in a pestle and mortar, pelletized, sealed in an evacuated quartz tube, and heated in an electric furnace. The heat treatment condition was 725 °C for 15 h for both samples. However, for La_2_O_2_Bi_3_Ag_0.6_Sn_0.4_S_6−*z*_Se_*z*_, heating the sample to 725 °C in 1 h was needed to suppress the generation of impurity phases. The obtained samples were reground for homogeneity, pelletized, and heated in the same procedure. The phase purity of the prepared samples and the optimal annealing conditions were examined using X-ray diffraction (XRD) with a Cu-K_α_ radiation. The lattice parameters were determined using the Rietveld method with RIETAN-FP^35^. Schematic image of the crystal structure was drawn using VESTA^36^. The actual composition was analyzed by energy-dispersive X-ray spectroscopy (EDX) on scanning electron microscope TM3030 (Hitachi). The magnetic susceptibility measurements were carried out using a superconducting quantum interference device (SQUID) magnetometer (MPMS-3, Quantum Design). The susceptibility data were taken after both zero-field cooling (ZFC) and field cooling (FC). The temperature dependence of electrical resistivity [*ρ*(*T*)] was measured by four-terminal method on the Physical Property measurement system (PPMS, Quantum Design). The resistivity measurement down to 0.4 K was measured using a ^3^He probe platform of PPMS. The ADR system on PPMS was used for resistivity measurements down to 0.1 K. For clarity, we labeled the examined samples with the nominal compositions. The Seebeck coefficient was measured by a four-probe method on ZEM-3 (Advance RIKO) at 300 K.

## Data Availability

The datasets generated and analyzed during the current study are available from the corresponding author on reasonable request.
